# Together throughout the year: seasonal patterns of bacterial and eukaryotic microbial communities in a macrotidal estuary

**DOI:** 10.1186/s40793-025-00664-y

**Published:** 2025-01-20

**Authors:** Vincent Hervé, Jérôme Morelle, Josie Lambourdière, Pascal Jean Lopez, Pascal Claquin

**Affiliations:** 1 Université Paris-Saclay, INRAE, AgroParisTech, UMR SayFood, 91120 Palaiseau, France; 2 Department of Biology and CESAM—Centre for Environmental and Marine Studies, University of Aveiro, Campus de Santiago, 3810-193 Aveiro, Portugal; 3 UMR BOREA, Muséum National d’histoire Naturelle, CNRS-8067, Sorbonne Université, IRD, Université de Caen Normandie, Université des Antilles, Paris, France; 4 Université de Caen Normandie, Laboratoire MERSEA UR 7482, Centre de Recherches en Environnement Côtier, 14530 Luc-sur-Mer, France

**Keywords:** Metabarcoding, Seine River, Longitudinal gradient, Season, Trophic interactions

## Abstract

**Background:**

Estuaries are complex ecosystems linking river and marine environments, where microorganisms play a key role in maintaining ecosystem functions. In the present study, we investigated monthly 8 sites at two depth layers and over a one-year period the bacterial and eukaryotic community dynamics along the Seine macrotidal estuary (Normandy, France). To date, the taxonomy of the microbial diversity present in this anthropized estuary remains elusive and the drivers of the microbial community structure are still unknown.

**Results:**

The metabarcoding analysis of 147 samples revealed both a high bacterial and eukaryotic diversity, dominated by *Proteobacteria, Bacteriodota, Actinobacteriota* and Bacillariophyta, Spirotrichea, Dinophyceae, respectively. Along the estuary we only detected significant spatial patterns in the bacterial and eukaryotic community compositions for three and two months out of twelve, respectively. However, we found a clear seasonal effect on the diversity of both microbial communities driven by physical and chemical variables that were fluctuating over the year (temperature, irradiance, river flow). Biotic associations were also significant drivers of both *alpha* and *beta* diversity. Throughout the year, we identified a diverse and abundant core microbiota composed of 74 bacterial and 41 eukaryotic OTUs. These regionally abundant species include habitat generalists encompassing heterotrophs, phototrophs and consumers. Yet, many of these core OTUs remain taxonomically and functionally poorly assigned.

**Conclusions:**

This molecular survey represents a milestone in the understanding of macrotidal estuary dynamics and the Seine ecosystem, through the identification of putative markers of ecosystem functioning. It also identifies seasons and biotic associations as main drivers of the Seine estuary microbiota and reveals the importance of a core microbiota throughout the year.

**Supplementary Information:**

The online version contains supplementary material available at 10.1186/s40793-025-00664-y.

## Background

Estuarine ecosystems, where freshwater from rivers meets and mixes with seawater, are among the most productive and dynamic environments on Earth [1]. These transitional zones exhibit unique physical, chemical, and biological characteristics that are critical for numerous ecological functions and services [2–5]. The interplay of various factors, including nutrient dynamics, hydrological regimes, and biological interactions, drives the ecological processes within estuaries, influencing their role in global biogeochemical cycles and as habitats for diverse communities of organisms [6, 7]. Estuaries are primarily physically dominated ecosystems where both vertical and horizontal gradients play crucial roles in structuring environments. The mixing of river freshwater and seawater in estuaries creates salinity, nutrient and turbidity gradients affecting many biological processes like primary production or organic matter recycling [8–11]. Additionally, estuarine gradients and circulation patterns vary significantly with tides and flow velocities, with seasonal changes strongly tied to the hydroclimatic setting such as precipitations [12]. These physicochemical gradients, horizontal, vertical, and temporal, contribute to the high diversity of habitat types within estuarine systems.

Microbial communities, encompassing both bacteria and eukaryotes, are fundamental to estuarine functioning. They play pivotal roles in nutrient cycling, organic matter decomposition, and primary production [1, 10, 13–15]. The composition, diversity, and activity of these microorganisms are influenced by various factors, ranging from salinity gradients and nutrient availability to anthropogenic impacts and climatic variations [2]. Understanding these microbial dynamics is essential for assessing the functioning of biogeochemical cycles [3] and sustainability of estuarine ecosystems. Microbial communities are key components in the trophic networks of estuarine systems including primary producers (phytoplankton, microphytobenthos), but also consumers, secondary producers or decomposers. Previous studies carried out in estuaries, have for example shown that the microbial communities dynamic differs due to the variability in physicochemical processes [16, 17]. Indeed, the strong gradients that apply in estuaries represent highly dynamic and species selective forces that cause temporal and spatial successions with rapid shifts in the microbial communities structure [17–22] leading to profound ecological changes [23]. Among studies that have characterized the role of physicochemical gradients on both bacterial and eukaryotic communities, each spatial and temporal gradient was found to play a significant role. For example, the vertical gradient of dissolved oxygen between the surface and the bottom layers in the Chesapeake Bay was shown to induce changes in microbial gene expressions [24]. The longitudinal abiotic gradients (salinity and silicates) mainly explained the variation in phytoplankton species composition of the Elbe and Schelde estuaries [25]. Nutrient pulses were proposed to be able to explain the phytoplankton community composition of the shallow estuary of Galveston Bay [26]. Similarly, the water temperature was identified as the main variable driving the microbial community shifts in the Pearl River estuary [27]. The vertical and spatial variabilities seems to be stronger than the temporal ones as shown for the bacterioplankton community composition across the river to ocean gradient of the Columbia River [28] although a weak correlation among samples to seasonal effect was shown in the estuaries of the mid-Atlantic states along the eastern coast of the USA [22]. However, while all of these gradients may be found within each estuary, differences between estuaries and locations also appear to be enough to explain the difference between the microbial communities [22, 25], highlighting the need to consider each ecosystem individually. The Seine estuary, a macrotidal system representing the largest estuarine complex in northwestern France and located at the outlet of the most anthropized watershed in the country, has been extensively studied, and its overall functioning is well understood [9–11]. However, little is known about the variations in microbial communities on a broad scale and how these variations align with the functioning of the estuary [29]. This gap underscores the necessity for more comprehensive studies that integrate microbial community dynamics into our understanding of such critical ecosystems.

In addition, changes in the microbial community can also result from biotic interactions, such as selective grazing [30], parasitism [31], mutualism [32] or even viral dynamics [33]. Drivers of specific associations between bacteria and microbial eukaryotes and especially phytoplankton include environmental factors such as (nutrient, light, temperature), as well as biotic interactions [34, 35]. These specific associations are mainly linked to the extracellular compounds produced by phytoplankton, which are rich in carbohydrates and represent a favorable environment for bacteria. These interactions, including the less specific ones such as competition, commensalism, or mutualism, that occur between these two biologic compartments consequently participate in biogeochemical cycling and play an important role in the microbial loop [36]. Despite the fact that it is not clear which factors control the interaction of bacteria and microbial eukaryotes, it is obvious that the strong physicochemical forcings that apply to estuarine microbial communities might play an important role in their biotic associations. However, to our knowledge, very few studies have investigated this topic (e.g. [37]), and especially in a highly anthropized hydrosystem [38].

In this context, the present study aimed to understand how physicochemical factors affect the bacterial and eukaryotic communities and their relationships in estuarine systems. This was achieved by examining changes in both the bacterial and eukaryotic microbial communities in relation to the main environmental factors in the temperate macrotidal estuary of the Seine River (France). Metabarcoding of 16S and 18S rRNA gene amplicons was used to characterize the whole microbial communities along both the horizontal and the vertical gradients of the estuary throughout a whole year. We hypothesized that physicochemical factors in the estuary, across horizontal, vertical, and temporal gradients, would significantly influence the structure and dynamics of bacterial and eukaryotic communities, leading to distinct spatial and temporal patterns in community composition and diversity. Additionally, we also hypothesized that these factors might modulate the associations between microbial communities, thereby shaping the structure and function of the microbial ecosystem. This study highlights for the first time the dynamics of the microbial communities in the Seine estuary and provides new insights into the links between microbial diversity and environmental conditions in a temperate and macrotidal estuarine system.

## Methods

### Site and sampling

**Fig. 1:**
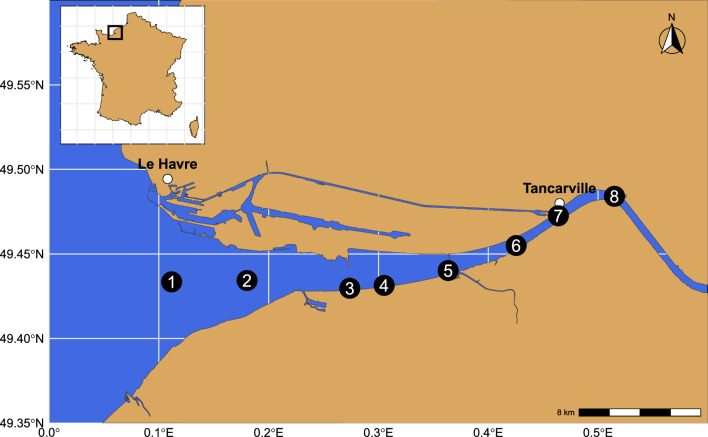
Localization of the eight sites sampled monthly for one year along the Seine estuary

The temperate macrotidal Seine Estuary, situated on the French coast of the English Channel, features a semidiurnal tidal range that reaches up to 8 m in its downstream part. It is one of the largest estuaries on the Northwestern European continental shelf, with a drainage basin exceeding 79,000 km^2^. The Seine River's flow varies from 100 to 2,300 m^3^ s^−1^ during low and high river flow periods, respectively, with an average annual flow of approximately 450 m^3^ s^−1^ calculated over the past 20 years [39]. The sampling was conducted monthly from January to December 2015, allowing consideration of the temporal gradient. The tide in this estuary is characterized by a prolonged period of high tide, lasting more than 2 h, due to the deformation of the tidal wave during its propagation in shallow depths [40, 41]. The irradiance (µmol photons m^−2^ s^−1^) at the water surface was obtained from the nearest national weather station (18 miles; 29 km) and the Seine River flow (m^3^ s^−1^) was obtained from continuous national measurements conducted at the Vernon station (source: HydroPortail). In order to consider the horizontal gradients of the estuary, eight sites distributed along the salinity gradient were sampled during the high tide phase of spring tides, ensuring that all sites were sampled under stable water conditions (Fig. 1). At each site, temperature (°C), salinity (PSU), and turbidity (NTU) were recorded in vertical profiles from the surface (-1 m) to the water–sediment interface (+ 1 m) using a probe SBE 19-PlusVD CTD (Seabird). Water samples were taken from the surface layer at each site (from 1 to 8) while the depth layer was sampled at four sites (2, 4, 6, and 8) allowing consideration of the vertical gradients. All samples were immediately filtered on a 500 µm mesh and stored before being used to estimate the concentrations in nutrients (N, P, Si), suspended particles matter (SPM), extracellular and transparent polymeric substances (EPS and TEP), and chlorophyll *a*. The photosynthetic parameters including the physiological state of the cells (F_V_F_M_), the relative maximal electron transport rate (rETR_max_) and the daily phytoplanktonic primary production estimates were calculated with fluorescence measurements performed using a water-EDF PAM fluorometer. All these environmental variables were presented in a previous study resulting from the same sampling effort and more details on sampling and methods are available in [10]. To characterize the microbial (bacterial-16S and eukaryotic-18S) communities, a 25 ml water sample from each site was successively filtered through 5 and 0.2-µm TMTP filters (Millipore, Merck), and immediately stored at −80 °C until DNA extraction.

### DNA extraction and library preparation

Total environmental DNA was extracted from the filters of each sample using a PowerBiofilm DNA Isolation kit according to the manufacturer’s procedure (MO BIO, Qiagen, CA, USA) as described in [42]. The V4-V5 region of the 16S rRNA gene region was amplified using the 515F-Y and 962R primers [43], and the V1-V3 region of the 18S rRNA gene region was amplified using the 18S_0067a_deg and NSR399 primers [44]. We first performed the PCR amplification using 1 μl of DNA (5–10 ng) in the following mix: 1 μl DNA, 1 μl forward Primer (10 μM), 1 μl reverse Primer (10 μM), 0.75 μl DMSO, 0.25 μl BSA (10x), 8.5 μl H2O and 12.5 μl PCR Master Mix 2x (KAPA2G Robust HotStart DNA polymerase ReadyMix, KAPA Biosystems, Sigma-Aldrich, France). The following amplification program was used: 95 °C for 5 min; 30 cycles of 95 °C for 15 s, 52 °C for 15 s, and 72 °C for 30 s; and 72 °C for 3 min. The PCR products were checked on an agarose gel, purified using Agencourt AMPure XP beads (Beckman Coulter, High Wycombe, UK), and quantified using a Qubit dsDNA HS assay kit. They were then normalized and pooled (2 pools). The libraries were prepared with 1 μg of the pooled DNAs and using the Illumina TruSeqDNA PCR-Free Library Preparation Kit (Illumina France SARL, San Diego, CA, USA). The supplier protocol was followed with the exception that a modified End-Repair mix was used to avoid the production of chimeric constructs. The resulting libraries were quantified by qPCR and sequenced using an Illumina MiSeq 2 × 300 paired-end run, by Fasteris SA (Plan-les-Ouates, Switzerland).

### Sequence processing

Amplicon sequences were analyzed with the *mothur* software version 1.39.1 [45], as previously described [46]. First, contigs between read pairs were assembled. Then, barcode and primer sequences and low-quality sequences were removed (minimum length of 300 bp, removing any sequences with ambiguous bases and removing any sequences with homopolymers longer than 8 bp). Subsequently, sequences were aligned to the SILVA SSU reference database [47] and preclustered (*pre.cluster*, diffs = 1). Singletons were excluded and chimeras were removed with *chimera.uchime* command in *mothur*. Then, sequences were classified using the naive Bayesian classifier [48] implemented in *mothur* with the SILVA reference database release 138 and the PR2 database version 4.12 [49] for the 16S and 18S rRNA gene reads respectively. After classification, non-bacterial, chloroplast, mitochondria (for the 16S rRNA gene dataset), non-eukaryotic (for the 18S rRNA gene dataset) and unknown (for both 16S and 18S rRNA amplicons) sequences were excluded. Archaeal sequences were excluded from the 16S rRNA gene dataset. A total of 2,600,102 (ranging from 7,004 to 42,209 reads per sample, median of 16,222) and 6,722,943 (ranging from 20,624 to 102,790 reads per sample, median of 41,976) reads were obtained for the 16S and 18S rRNA gene datasets, respectively. To account for differences in sampling efforts, 7,004 and 20,624 sequences from the 16S and 18S rRNA gene datasets respectively, were then randomly subsampled from each sample [50]. OTUs were generated using the *OptiClust* algorithm [51], with an OTU being defined at the 97% and 99% sequence similarity level for the 16S and 18S rRNA gene reads respectively. The raw sequence data have been deposited in the NCBI Sequence Read Archive under the BioProject PRJNA1164255.

### Diversity and statistical analyses

*Alpha* diversity indices were computed with *mothur*. All the other analyses were performed with R software version 4.3.1. Data manipulation and visualization was done with *tidyverse* collection of R packages [52]. The map was generated with the *sf* package [53].

Random forest models were built to evaluate the relative importance of biotic (bacterial or eukaryotic richness) and abiotic (sampling site, latitude, longitude, depth, season, flow, irradiance, temperature, salinity, N, P, Si, SPM, Chla, EPS, TEP) factors on the microbial richness, with the *rfPermute* package using 10^4^ trees and 10^4^ permutations. Mantel and partial Mantel tests were computed with the *ecodist* package [54], based on Pearson correlations, with 10⁶ permutations, using Bray–Curtis distances for the microbial communities and Euclidean distances for geographical, temporal and physico-chemical matrices. Both nonmetric multidimensional scaling (NMDS) and redundancy analysis (RDA) were generated with *vegan* [55] and *ggordiplots* packages. PERMANOVA were computed based on 10^4^ permutations using *vegan*. To perform the RDA, a Hellinger transformation was applied to the community matrices and the environmental variables were standardized. Only statistically significant variables (*P* < 0.05) were retained using forward selection. The significance of the RDAs was tested with 10^4^ permutations. Partitioning of the *beta* diversity was performed with the *betapart* package and using the Jaccard family index [56].

The core microbiota of each microbial community was defined with a minimum prevalence of 0.8 [57], *i.e.* all the OTUs present in at least 80% of the samples and thus covering the four seasons and the 12 sampling sites (8 in surface and 4 in lower layers). The heatmap of the core microbiota was generated with the *mixOmics* package [58] with the average (UPGMA) clustering method. Prior to clustering, OTU abundances were standardized using centered-log ratio transformation. Potential function among bacteria was predicted by using FAPROTAX v1.2.7, which was initially designed for marine samples [59]. For eukaryotes, functional groups were assigned using trophic groups [60].

## Results

**Fig. 2:**
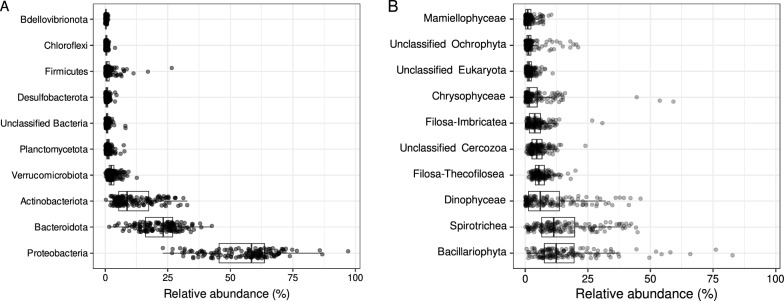
Overview of the taxonomic distribution of the ten major (**A**) bacterial phyla and (**B**) eukaryotic clades (rank-2 level based on the PR2 classification system) present in the 147 samples. Each point represents the relative abundance (% of the number of reads) of each clade in a sample

Over a year, eight sites were sampled monthly in the Seine estuary (Fig. 1) and various environmental variables were measured (Table S1, for details see [10]). For temperature, the range was from 5.1 to 21.9 °C (median 13.6) while salinity varied between 0.3 and 29.9 PSU (median 14), flow varied between 181 and 1,070 m^3^ s^−1^ (median 328) and irradiance varied between 225 and 2,064 µmol photons m^−2^ s^−1^ (median 1,123). A total of 147 samples, composed of 100 surface and 47 deeper layer samples, were successfully amplified and sequenced for both 16S and 18S rRNA gene markers. This molecular survey of the Seine estuary revealed the presence of 11,546 bacterial OTUs (median 656 OTUs per sample) and 27,055 eukaryotic OTUs (median 1,030 OTUs per sample). Among the bacterial communities, we identified 63 phyla dominated by *Proteobacteria* (median relative abundance 58.5%), *Bacteroidota* (23.2%), *Actinobacteriota* (8.75%), *Verrucomicrobiota* (2.36%) and *Planctomycetota* (1.16%). Among the eukaryotic communities, 157 rank-2 clades were identified using the PR2 system, dominated by Bacillariophyta (diatoms, median relative abundance 12.4%), Spirotrichea (11.4%), Dinophyceae (5.98%), Filosa-Thecofilosea (5.41%), unclassified Cercozoa (4.58%), Filosa-Imbricatea (3.76%), Chrysophyceae (1.53%) (Fig. 2).

**Fig. 3:**
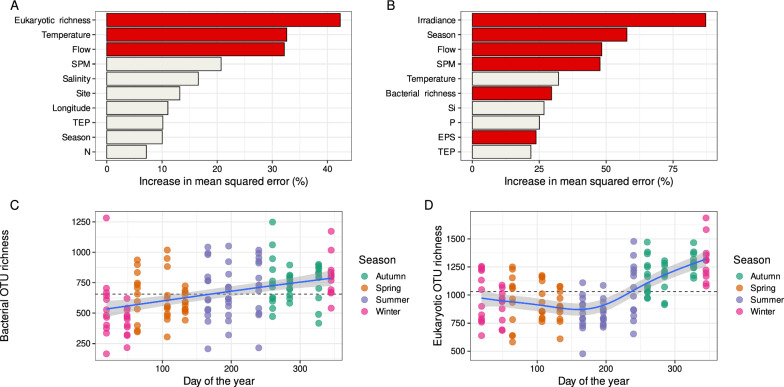
Microbial OTU richness. Mean predictor importance (% of increase of mean squared error) of environmental drivers on microbial OTU richness for (**A**) the bacteria and (**B**) the eukaryotes. Only the variables represented by red barplots are significant (*P* < 0.05). Temporal evolution of the (**C**) bacterial and (**D**) eukaryotic richness across the year. The horizontal black dashed line represents the median richness of the whole dataset. The blue line represents a regression fitted using the “gam” method. Shaded areas correspond to the point wise 95% confidence interval on the fitted values. SPM: suspended particulate matter; TEP: transparent polymeric substances; EPS: extracellular polymeric substances

Several significant predictors of microbial richness were found, encompassing both biotic and seasonal variables (Fig. 3). Random forest models indicated that eukaryotic richness, temperature and river flow were the major predictors of bacterial richness (Fig. 3A). Regarding the eukaryotic richness, irradiance, season, flow, suspended particulate matter (SPM), bacterial richness and exopolysaccharides (EPS) were the main significant predictors (Fig. 3B). The importance of the interaction between the microbial communities was confirmed by the significant and positive relationship between bacterial and eukaryotic richness (*r* = 0.33, *P* < 0.001). Concerning the seasonal effect, microbial richness significantly changed throughout the year, following a positive and linear trend for the bacteria (Fig. 3C) while eukaryotic richness tended to slightly decrease during the first half of the year and then to significantly increase during the second half (Fig. 3D). Noteworthy, throughout the year no significant spatial predictor (latitude, longitude, and depth) for the bacterial or eukaryotic richness were found.

The *beta* diversity of the microbial communities was investigated through different approaches. First, Mantel and partial Mantel tests were used to identify the main drivers of community composition for the whole dataset (Table 1). Bacterial community composition was strongly correlated to the eukaryotic community composition (r_M_ = 0.642, *P* < 0.001), even when accounting for physicochemical variables, spatial and temporal distances (r_M_ = 0.626, *P* < 0.001). Temporal distance had a significant effect on both microbial communities but to a lesser extent when accounting for physico-chemical variables, spatial and microbial distances (r_M_ = 0.075. and r_M_ = 0.34, for bacteria and eukaryotes, respectively). No significant relationship was found between the geographic distances and the microbial community compositions (*i.e.* no distance-decay relationship). Physicochemical variables had a significant effect on both microbial community compositions, but this effect disappeared for eukaryotes when considering bacterial, spatial and temporal distances. Additionally, environmental variables (*i.e.* physicochemical variables) significantly changed temporally (r_M_ = 0.399, *P* < 0.001) but not spatially (r_M_ = 0.023, *P* > 0.14) (Table 1). Similar trends were identified when considering surface or deep layer samples independently (Table S2). Since no distance-decay relationship was detected throughout the year, a monthly analysis was performed for both bacterial and eukaryotic communities. For the twelve months, the distance-decay relationship was only detected for three months for the bacterial communities (August, October, December) and for two months for the eukaryotic communities (October, December). In all these five cases, the Mantel correlation coefficient between the bacterial and eukaryotic matrices (biotic association) was always higher than the correlation coefficient between the bacterial or eukaryotic matrix and the spatial distance (distance-decay relationship). For these five cases, we also identified a significant and strong correlation between environmental variables and geographical distances, confirming that environmental variables changed along the estuary and suggesting that these changes contribute to changes in microbial diversity patterns (Table S2).

**Table 1:** Mantel and partial Mantel tests based on Pearson correlations, with 10⁶ permutations, using Bray–Curtis distances for the microbial communities and Euclidean distances for spatial (latitude, longitude, depth), temporal (day of the year) and physicochemical (flow, irradiance, temperature, salinity, N, P, Si, SPM, Chla, EPS, TEP) matrices. The physicochemical matrix is called Chemistry in the table

	**r**_**M**_	***P* value**
**Bacteria ~ Eukaryotes**	**0.642**	** < 0.001**
**Bacteria ~ Eukaryotes + Chemistry + Space + Time**	**0.626**	** < 0.001**
**Bacteria ~ Chemistry**	**0.231**	** < 0.001**
**Bacteria ~ Chemistry + Eukaryotes + Space + Time**	**0.130**	** < 0.001**
Bacteria ~ Space	-0.009	0.672
Bacteria ~ Space + Chemistry + Eukaryotes + Time	-0.011	0.708
**Bacteria ~ Time**	**0.202**	** < 0.001**
**Bacteria ~ Time + Space + Chemistry + Eukaryotes**	**0.075**	** < 0.001**
**Eukaryotes ~ Bacteria**	**0.642**	** < 0.001**
**Eukaryotes ~ Bacteria + Chemistry + Space + Time**	**0.623**	** < 0.001**
**Eukaryotes ~ Chemistry**	**0.159**	** < 0.001**
Eukaryotes ~ Chemistry + Bacteria + Space + Time	0.001	0.469
Eukaryotes ~ Space	-0.003	0.559
Eukaryotes ~ Space + Chemistry + Bacteria + Time	0.004	0.390
**Eukaryotes ~ Time**	**0.158**	** < 0.001**
**Eukaryotes ~ Time + Space + Chemistry + Bacteria**	**0.034**	**0.027**
Chemistry ~ Space	0.023	0.148
**Chemistry ~ Time**	**0.399**	** < 0.001**

Significant relationships are highlighted in bold

**Fig. 4:**
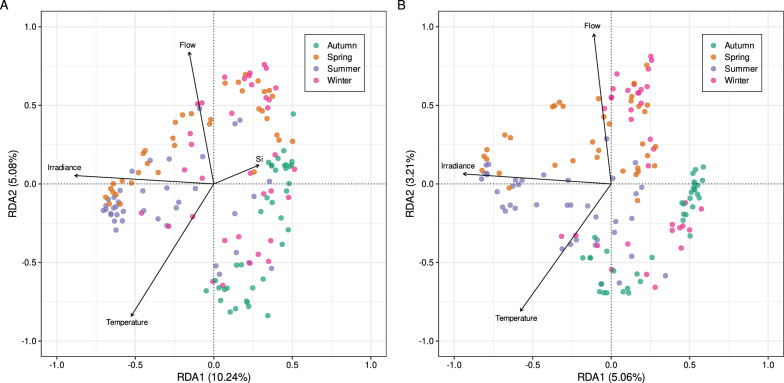
Correlation biplot based on a RDA ordination of the (**A**) bacterial and (**B**) eukaryotic communities constrained by the environmental variables. Only the environmental variables retained by forward selection (*P* < 0.05) are presented

Second, unconstrained ordinations were applied (NMDS; Figure S1), which identified seasons as a significant driver of the microbial *beta* diversity (PERMANOVA, R^2^ = 0.175, *P* < 0.001 and R^2^ = 0.082, *P* < 0.001; for bacterial and eukaryotic communities, respectively). No significant difference was observed between composition of surface and deep layer communities (PERMANOVA, R^2^ = 0.003, *P* = 0.953 and R^2^ = 0.004, *P* = 0.947; respectively for bacterial and eukaryotic communities), suggesting the absence of vertical stratification during sampling. Then canonical ordinations through redundancy analysis were applied on both community matrices (Fig. 4), which showed significant models (adjusted R^2^ = 0.141, *P* < 0.001 and adjusted R^2^ = 0.072, *P* < 0.001 for bacterial and eukaryotic communities, respectively). Irradiance, temperature and river flow were identified as significant explanatory variables for both communities (*P* < 0.005). Noteworthy, these three variables are known to vary along the year following a seasonal pattern. Additionally, Si content also had a significant impact but only on the bacterial community composition (variance = 0.007, *P* = 0.017). Although the two first axes of the RDAs (mainly driven by temperature, irradiance, and flow) only explained respectively 15.32% and 8.27% of the bacterial and eukaryotic community variation, a seasonal effect was observable, with a clear separation between samples from autumn and spring for both microbial communities (Fig. 4). At a finer scale, this temporal effect was also clear when considering day of sampling instead of the season (Figure S2).

Third, the *beta* diversity was partitioned, showing that turnover, *i.e.* species replacement, was predominantly driving the *beta* diversity (median values 94.92% and 98.42% for bacteria and eukaryotes, respectively) while nestedness had a very minor contribution (median values 5.08% and 1.58% for bacteria and eukaryotes, respectively) (Figure S3).

**Fig. 5:**
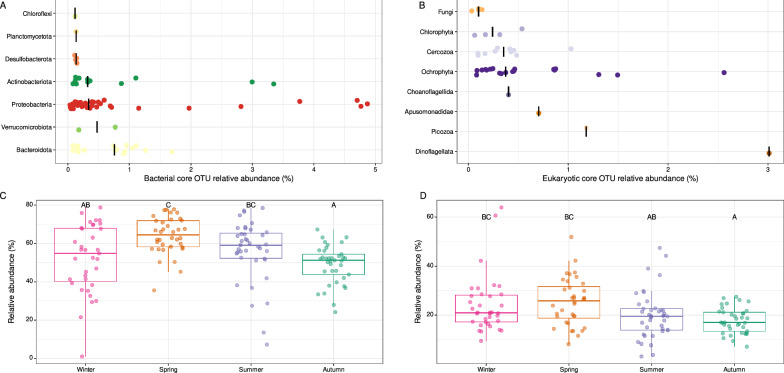
Core microbiota. Taxonomic distribution and mean relative abundance of the core OTUs of the (**A**) bacterial and (**B**) eukaryotic communities. Vertical black lines correspond to median values. Seasonal variations of the relative abundance of core microbiota for (**C**) the bacterial and (**D**) the eukaryotic communities. Statistical comparisons were computed with Kruskal–Wallis test followed by Dunn’s test with Bonferroni adjustment

**Fig. 6:**
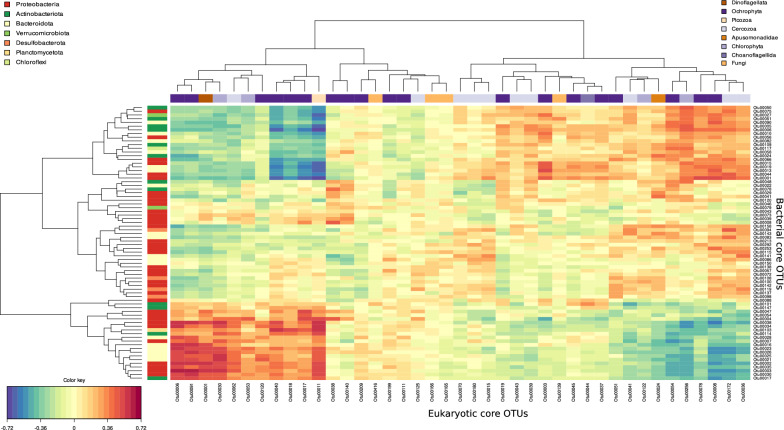
Core microbiota. Heatmap showing the Pearson correlations between the core bacterial and core eukaryotic OTU abundances. The color code for OTU taxonomy is similar to the one used for Fig. 5. Refer to Table S3 for the complete taxonomic assignment of these OTUs

The core microbiota, defined by all the OTUs present in at least 80% of the samples, was extracted for further analyses. A total of 74 bacterial OTUs (0.64% of the identified bacterial OTUs) encompassing 55.33% of the total bacterial relative abundance and 41 eukaryotic OTUs (0.15% of the identified eukaryotic OTUs) representing 21.80% of the total eukaryotic relative abundance was identified. These OTUs of the core microbiota present a relatively high relative abundance throughout the year, ranging from 0.03 to 4.87% in the dataset (Fig. 5A and B). Interestingly, significant seasonal variations were observed for the relative abundance of the core microbiota for both bacterial and eukaryotic communities (Kruskall-Wallis, *P* < 0.05) with lower relative abundances in autumn than in spring for both communities (Dunn’s test, *P* < 0.05) (Fig. 5C and D). This core microbiota was also phylogenetically diverse with seven bacterial phyla including 36 *Proteobacteria*, 18 *Bacteroidota* and 12 *Actinobacteriota* and eight PR2-rank3 eukaryotic clades that included 14 diatoms (Bacillariophyta, Ochrophyta), 10 Cercozoa, four Chlorophyta, four Fungi, one Picozoa (genus *Picomonas*) and a Dinoflagellata (genus *Heterocapsa*) (Table S3). Positive and negative correlations were observed between the relative abundances of the bacterial and eukaryotic OTUs (Fig. 6). In particular, a clear cluster of highly positively correlated OTU abundances (Fig. 6, bottom left) was found between the bacterial (*Flavobacteriaceae* NS5 and NS4 marine groups, Candidatus *Actinomarina*, Candidatus *Puniceispirillum, Planktomarina, Methylophilaceae* OM43 clade, SAR11 clade) and eukaryotic (*Heterocapsa*, *Thalassiosira, Ostreococcus, Picomonas*) communities. A second cluster of highly positively correlated OTU abundances was identified (Fig. 6, top right), dominated by different bacteria (*Limnohabitans, Sporichthyaceae* hgcI clade, *Alcaligenaceae* GKS98 freshwater group, *Polynucleobacter, Flavobacterium*) and eukaryotes (Chrysophyceae clade C, *Reckertia*, Sphaeropleales, two centric diatoms, two Cercozoa) (Table S3).

## Discussion

### Taxonomic diversity in the Seine estuary

The results presented in this study highlight the remarkable microbial diversity within the Seine estuary that was never described at such a level, especially regarding the spatial and temporal scales. They expand on recent results from the Seine rivers sampled in the Parisian region [61, 62]. A total of 147 samples, comprising both temporal and spatial samples with surface and deeper layers, were successfully amplified and sequenced for both 16S and 18S rRNA gene markers, revealing a rich and complex microbial community. Specifically, 11,546 bacterial OTUs and 27,055 eukaryotic OTUs were identified, demonstrating the extent of microbial diversity within this estuarine system. Among the eukaryotic OTUs, a diverse array of functional roles can be associated to this community, encompassing both autotrophs and heterotrophs (Fig. 2, Table S3). The autotrophs were mainly represented by Bacillariophyta, considered as the main contributor to the global phytoplankton primary production in Seine estuary [10, 11, 63] but also by Dinophyceae and Chrysophyceae. Heterotrophs included predators and decomposers mainly represented by Spirotrichea (Ciliophora) known to dominate estuarine ciliate planktonic communities [64, 65] where they consume bacteria, diatoms, and dinoflagellates, and Filosa-Imbricatea (Cercozoa), already identified dominant in benthic heterotrophic protist communities [66], which represent important consumers of prokaryotes attached to particles in the sediment or suspended in water [67]. This suggests that the eukaryotic microbial community seems to be sufficiently diversified to fulfill the spectrum of eukaryotic functions and ecological roles required for stability, resilience, and ecosystem functionality within an estuarine environment [68]. Indeed, the carbon cycling occurring in estuaries is mainly represented through the exchanges among a complete set of trophic levels including microbial autotrophic primary producers, heterotrophic detritivores, fungi, and a diverse assemblage of predatory protists [68].

Regarding the 11,546 bacterial OTUs identified, the dominance of *Proteobacteria* aligns with their well-known ubiquity in estuaries [13, 69] and metabolic versatility in various aquatic environments [70, 71]. The high abundance of *Bacteroidota* and *Actinobacteriota* is consistent with the recent result of Xian et al. [72] where they were identified among the main contributors in mixed waters of the Yangtze River estuary. Known for their role in organic matter degradation, this result indicates their potential importance in nutrient cycling and organic matter decomposition in the estuary. The presence of *Verrucomicrobiota* and *Planctomycetota*, although less abundant, may indicate their specific ecological roles within the ecosystem, potentially related to carbohydrate degradation [73]. However, it is important to note that despite the comprehensive taxonomic coverage provided by the analysis, certain important bacterial groups such as *Cyanobacteria* seem to be underrepresented in the results. Although this could be potentially explained by sampling strategy, *i.e.* exclusion during the filtration step, *Cyanobacteria* from the pico- and nano-phytoplankton were also previously shown in very low abundances in this estuary [11, 74]. Serre-Fredj et al. [11], showed through cytometry measurements, extending even further upstream in the estuary than our study, that *Cyanobacteria* abundances were low throughout the estuary. Only summer peaks can be measured very far upstream, outside our study area. These observations can be explained by the fact that these *Cyanobacteria* (e.g. *Synechococcus*) preferentially develop in the upper part of well-lit euphotic zones, which is not the case in an ecosystem as dynamic and turbid as a macrotidal estuary [75]. *Cyanobacteria* abundance and diversity could also be strongly regulated by the presence of cyanophages in the estuary [76].

### Drivers of the community structure

Besides taxonomic diversity, we also aimed at identifying the drivers of microbial community structure in the Seine estuary. Contrary to our initial hypothesis on spatial variation, spatial pattern for both *alpha* (Fig. 3) and *beta* (Table 1, Fig. 4) microbial diversity was limited. Over the course of the year, we found no effect of depth nor geographical distance along the estuary. This result represents a notable finding, as it contrasts with the distributional patterns observed in other estuarine systems [77, 78]. Decomposing our dataset in monthly analyses revealed the existence of spatial patterns (*i.e.*, distance-decay relationship) for both bacterial and eukaryotic communities but only for a few months (2 for eukaryotes, 3 for bacteria) (Table S2). Interestingly, these two months (October and December) were common for bacteria and eukaryotes, suggesting a common behavior that is also supported by a positive and significant correlation between these two community matrices during these months (Table S2). Besides biotic associations, these spatial patterns can also be explained by the variations in physical and chemical variables along the estuary, as indicated by the high and significant correlations between these variables and the geographical distances. A potential explanation for this limited presence of spatial patterns might be due to a continuous mixing of the waters and to the intense and complex dynamics of environmental parameters within the macrotidal nature of this part of the Seine estuary [79]. Estuarine environments are indeed characterized by complex hydrodynamic processes, which can lead to rapid and unpredictable changes in environmental conditions. The dynamic nature of such estuarine systems introduces a complex interplay of physical, chemical, and biological factors, making it challenging to always discern clear and reproducible spatial patterns. The significant mixing of various water masses and the blending of different horizontal gradients (upstream–downstream: salinity, downstream-upstream: turbidity, nutrients) as well as vertical gradients might influence microbial communities in intricate ways, potentially masking the spatial patterns of diversity that might be observed in more stable or less dynamic environments. Additionally, the rapid turnover of environmental conditions within the estuary may lead to a lack of consistent spatial relationships between microbial diversity and environmental gradients. These intense dynamics were assumed to also explain the absence of vertical patterns observed. Thereby, while the Seine estuary is characterized by diverse spatial gradients in physical and chemical conditions [10, 11, 74], the limited spatial structuring, horizontally and vertically, in microbial communities underscores the dynamic nature of microbial communities in macrotidal estuarine systems, which exhibit here more temporal than spatial fluctuations.

Indeed, in accordance with our hypothesis on temporal variation, both *alpha* and *beta* diversity were significantly correlated with physicochemical variables which were in turn, correlated with time (Table 1, Figure S2), hence showing a seasonal pattern for both the bacterial and eukaryotic community assembly. Impact of seasons on microbial communities has already been observed in various estuaries [80, 81]. During the sampled year, we observed significant differences in microbial richness (Fig. 3) and microbial community composition (Fig. 4, Table 1, Figure S2), indicating a seasonality in the microbial community assembly of the Seine estuary. Similarly, we observed significant seasonal variations of the abundance of the core microbiota (Fig. 5). The influence of seasons on microbial community composition is widely acknowledged and has already been reported in urban [82, 83] and natural rivers [84], as well as in coastal waters [85].

Among the physicochemical variables measured here, flow, temperature, irradiance had the most significant influence on the *alpha* and *beta* diversity, and these three variables are known to fluctuate with seasons. They have also been reported as drivers of the distribution and co-occurrence of estuarine microbial groups [17]. Various studies have identified other environmental factors with significant influence on estuarine microbial communities such as salinity, hydrodynamics, and granulometry in English estuaries [86]; dissolved oxygen and salinity in Patagonian fjords [87]; temperature and salinity in an Arctic estuarine system [88]; and nutrient concentration and dissolved oxygen in the highly urbanized Sydney Harbor [13]. Overall, our findings are consistent with the broader understanding that stream microbial diversity can be modulated by environmental changes [89] and that the relative amount of community variation that is explained by environmental variables in estuaries is known to be moderate [90]. Environmental conditions within the estuary, including flow, temperature, irradiance and nutrients could further modulate the microbial diversity as well as the strength and nature of biotic associations, as it was already reported in the Skidaway river estuary [91].

Among all the measured variables, we identified biotic variables as the most important ones in explaining the community structure, regardless of the influence of time, space or physicochemistry (Table 1). Significant correlation between bacterial and eukaryotic diversity were identified for both *alpha* and *beta* diversity metrics. These correlations suggest strong biotic associations between bacterial and eukaryotic communities within the Seine estuary, shaping microbial dynamics in this ecosystem. The positive correlation between eukaryotic and bacterial richness is in line with the observation of Zhang et al. [92] showing that many phytoplankton species had positive co-occurrences with bacterial species. This suggests potential synergy or cooperative relationships between microbial groups that may involve nutrient exchange, habitat modification, or other forms of cooperation [68]. Similarly, positive and negative associations between bacteria and protists have been identified, for example within estuarine microbial networks [91]. These findings highlight the complexity of biotic associations within the estuarine ecosystem, potentially influencing ecosystem dynamics and functioning, including nutrient cycling.

### Importance of the core microbiota

The importance of biotic interactions was also pointed up by the presence of a core microbiota [93] in the Seine estuary (Fig. 5, Table S3). This core microbiota composed of 74 bacterial and 41 eukaryotic OTUs corresponded to a very minor fraction of the total richness. Yet this number of OTUs can be seen as relatively important when considering a dataset encompassing both spatial and temporal scales but also when taking into account the open and dynamic nature of the estuarine ecosystem [94]. This limited number of species can thereby be explained by the complexity of the macrotidal estuarine system studied [10], characterized by diverse environmental factors and fluctuations that impose selective pressures on the microbial communities. This rigorous selection process might have promoted the prevalence of species that are capable of adapting and thriving under diverse and sometimes challenging conditions, contributing to the observed low diversity but high abundance within the core microbiota [95]. Indeed, despite the low richness, a high abundance of the core microbiota was observed in this study representing more than 55% of the total relative abundance for bacteria and 21% for eukaryotes. Although definition of abundant taxa can vary, with more than 0.3% mean relative abundance for each member of the core microbiota (Fig. 5), all these core OTUs could be defined as regionally abundant OTUs [96]. Even though seasonal variations of the core abundance were detected (Fig. 5), the prevalence and abundance of this core microbiota would indicate that these 115 OTUs could be seen as habitat generalists [97]. In fact, among them certain clades have already been reported to have wide distribution in aquatic environment such as the bacteria *Planktomarina* [98], *Halieaceae* [99], *Chloroflexi* SL56 cluster [100], the diatoms *Thalassiosira* [101], the picoeukaryote *Picomonas judraskeda* [102] and the yeast *Malassezia globosa* [103]. Although these are broad categories, members of the core microbiota encompass various trophic levels, including photoheterotrophy, chemohetetrophy for bacteria as well as phototrophy, chemohetetrophy and consumers for eukaryotes (Table S3). Among the core bacteria, we also identified putative sulfate reducers belonging to SEEP-SRB1 *Desulfosarcinaceae* and *Desulfocapsaceae* [104, 105]. Regarding carbon utilization, two bacterial methylotrophs belonging to *Candidatus* Methylopumilus and OM43 clade [106, 107] were also present in the core microbiota. Among the eukaryotic core, chemoheterotrophs were represented by four fungal OTUs and one OTU assigned to *Picomonas judraskeda* [108] (Table S3). Phototrophs were dominated by 14 diatoms OTUs (Bacillariophyta), four Chlorophyta and one Heterocapsa (Dinoflagellata) while consumers were composed of four Cercozoa and one Apusozoa. Noteworthy, for both bacteria and eukaryotes, various OTUs could not be assigned to any trophic level or putative functional group, highlighting the need to further study these core species. Indeed, because of their abundance and prevalence, members of this core microbiota represent targets of choice to further investigate microbial metabolism as well as the contribution of these microorganisms to this macrotidal ecosystem functioning. One challenge could also be to look for the distribution and metabolic activity of the core microbiota at different periods during the day and at different tide periods. Future research should also include both culture-dependent, *e.g.* isolation of strains [109] for genome sequencing and physiological characterization, and culture-independent approaches *e.g.* metagenomics [110] to further reconstruct metagenome-assembled genomes (MAGs) [111–114] coupled with metatranscriptomic surveys [115]. Additionally, since many core OTUs could not be classified at the genus or species level, these genome-centric analyses would help to resolve their taxonomic assignment.

Our analysis of the core microbiota revealed clusters of positive and negative correlations between OTU relative abundances (Fig. 6). Species co-occurrences, inter- and intra-domain associations have been reported in various estuaries and urban waters [46, 92, 116–118]. Although these correlations do not always imply biological interactions between organisms [119], they suggest potential ecological relationships and functional associations between the Seine estuary core OTUs. The bottom left cluster of Fig. 6 was mainly composed of diatoms (Ochrophyta) positively correlated with members of *Proteobacteria* (*n* = 10), *Bacteroidota* (*n* = 6) and *Actinobacteriota* (*n* = 2) which correspond to phyla frequently found associated with diatoms in cultures or field samples [120, 121]. On the other hand, these diatom abundances were negatively correlated with the abundance of other *Proteobacteria* (*n* = 6), *Bacteroidota* (*n* = 6) and *Actinobacteriota* (*n* = 6) (left cluster of Fig. 6), suggesting host specificity between diatoms and bacteria [122]. Cercozoa were the second richest subdivision (*n* = 10) among the core eukaryotes after Ochrophyta (*n* = 18, including *n* = 14 Bacillariophyta or diatoms) and they were found in various abundance clusters. However, since some of the sequences could not be assigned to a low taxonomic rank, their identity and putative function remain elusive, highlighting the knowledge gap for this clade in the estuary ecosystem. Indeed, Cercozoa can encompass phototrophic, consumer and parasitic protists [60]. Here we could only taxonomically assign two OTUs to Filosa-Imbricatea and two others to Filosa-Thecofilosea, both clades described as consumers. Protist consumers are known to be abundant and important heterotrophs in estuaries [123, 124] and their presence in the core microbiota confirms their importance in the trophic network of the Seine estuary.

The presence of both positive and negative correlations suggests the existence of complex ecological interactions and regulatory mechanisms shaping microbial community structure and composition within the Seine estuary. Understanding this interplay is crucial for unraveling the functioning and resilience of the Seine estuarine ecosystem. Further research is warranted to elucidate the specific ecological roles and mechanisms underlying these associations and their implications for ecosystem health and resilience. Because the importance of seasonal physicochemical variables (temperature, irradiance, flow) was also clearly detected, this future work is crucial in the context of climate change, where extreme weather events are becoming increasingly frequent, posing potential disruptions to microbial communities, and undermining the resilience of estuarine ecosystems.

## Conclusions

Our molecular survey of the bacterial and eukaryotic communities along both a spatial and temporal gradient revealed for the first time a high microbial diversity in the Seine estuary. Against expectations, we only detected distance-decay relationships for the microbial communities for a few months and these spatial patterns were also associated with physicochemical and biotic variations. Throughout the year, we found temporal patterns of community diversity reflecting monthly and seasonal dynamics, again in relation with the physical and chemical variables of this macrotidal estuary. Besides these seasonal patterns, biotic associations emerged as a major driver of the microbial community structure. In particular, the identification of a diverse and abundant core microbiota composed of habitat generalists encompassing heterotrophs, phototrophs and consumers represents a breakthrough in the understanding of the functioning of this ecosystem. It also provides the bases for the biodiversity monitoring of this estuary as well as relevant species target for metabolic investigations.

## Supplementary Information

Additional file 1

Additional file 2

Additional file 3

Additional file 4

Additional file 5

Additional file 6

## Data Availability

Sequence data that support the findings of this study have been deposited in the NCBI Sequence Read Archive with the primary accession code PRJNA1164255.
