# Supplementary figures and images for: Together throughout the year: seasonal patterns of bacterial and eukaryotic microbial communities in a macrotidal estuary
Source: Environ Microbiome. 2025 Jan 20;20:8. doi: 10.1186/s40793-025-00664-y (PMC11748528; doi:10.1186/s40793-025-00664-y)

A

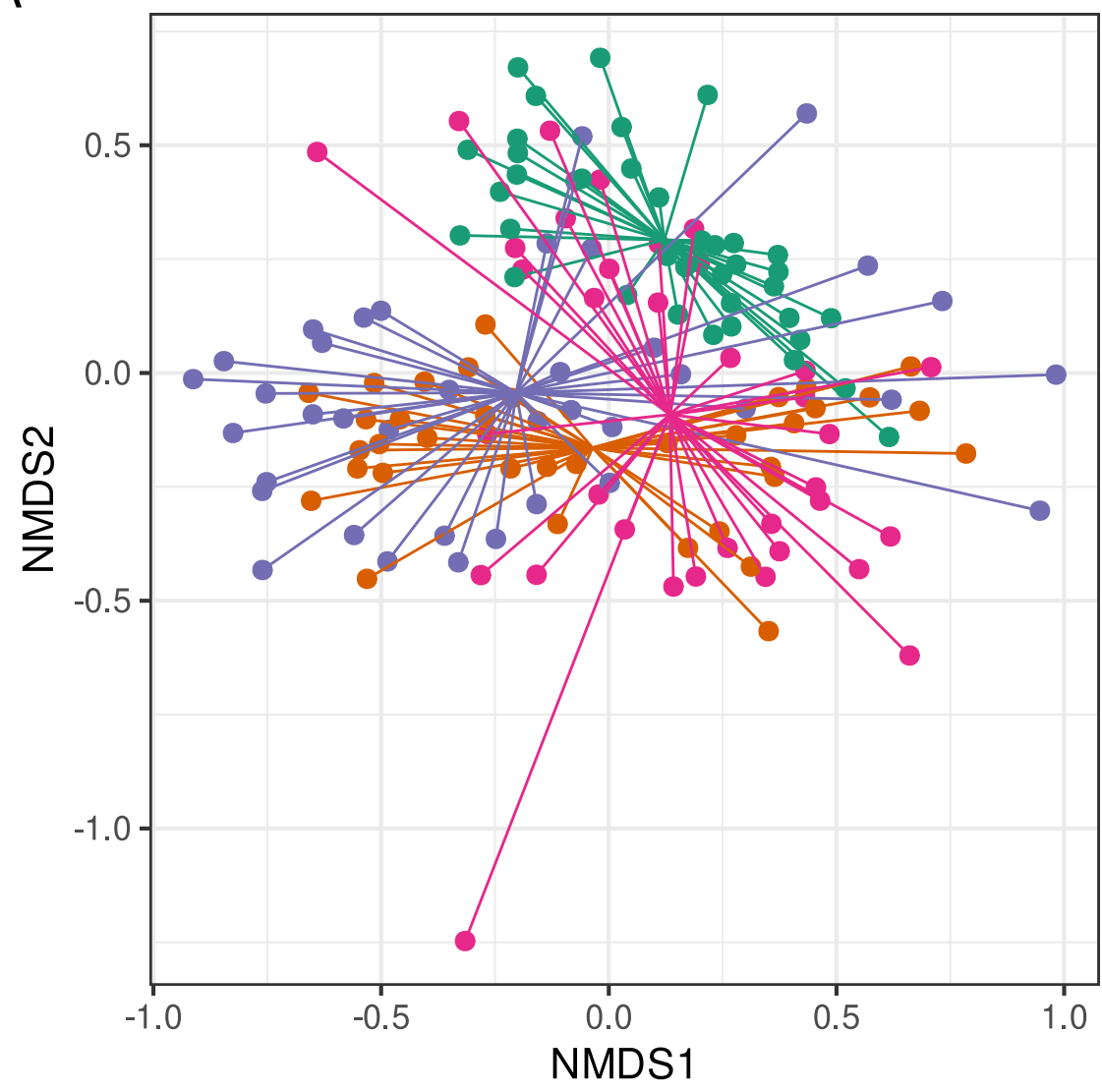

- Autumn
- Spring
- Summer
- Winter

B

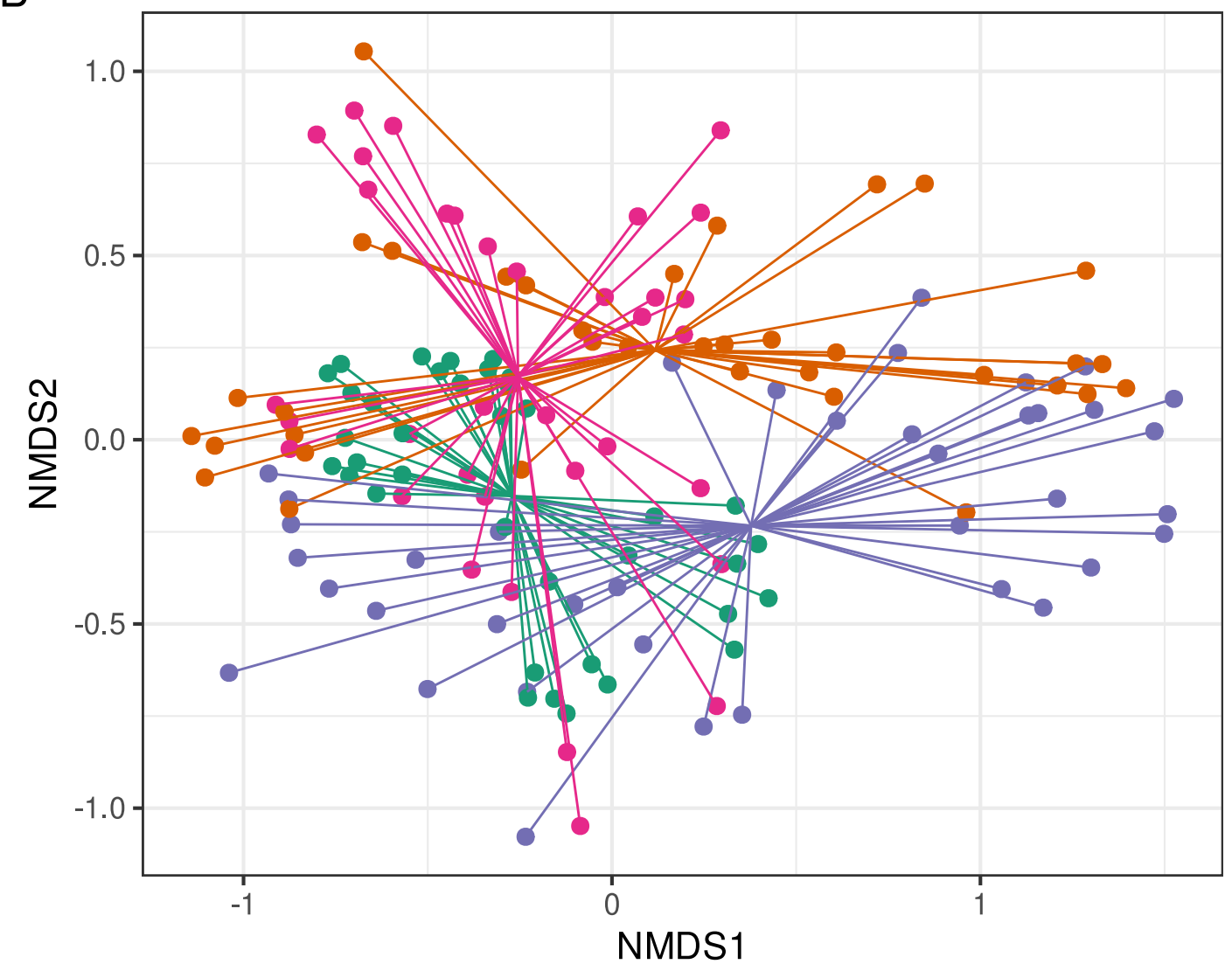

- Autumn
- Spring
- Summer
- Winter

Supplement: Supplementary file 1 — Additional file 1 [file 40793_2025_664_MOESM1_ESM.pdf]

A

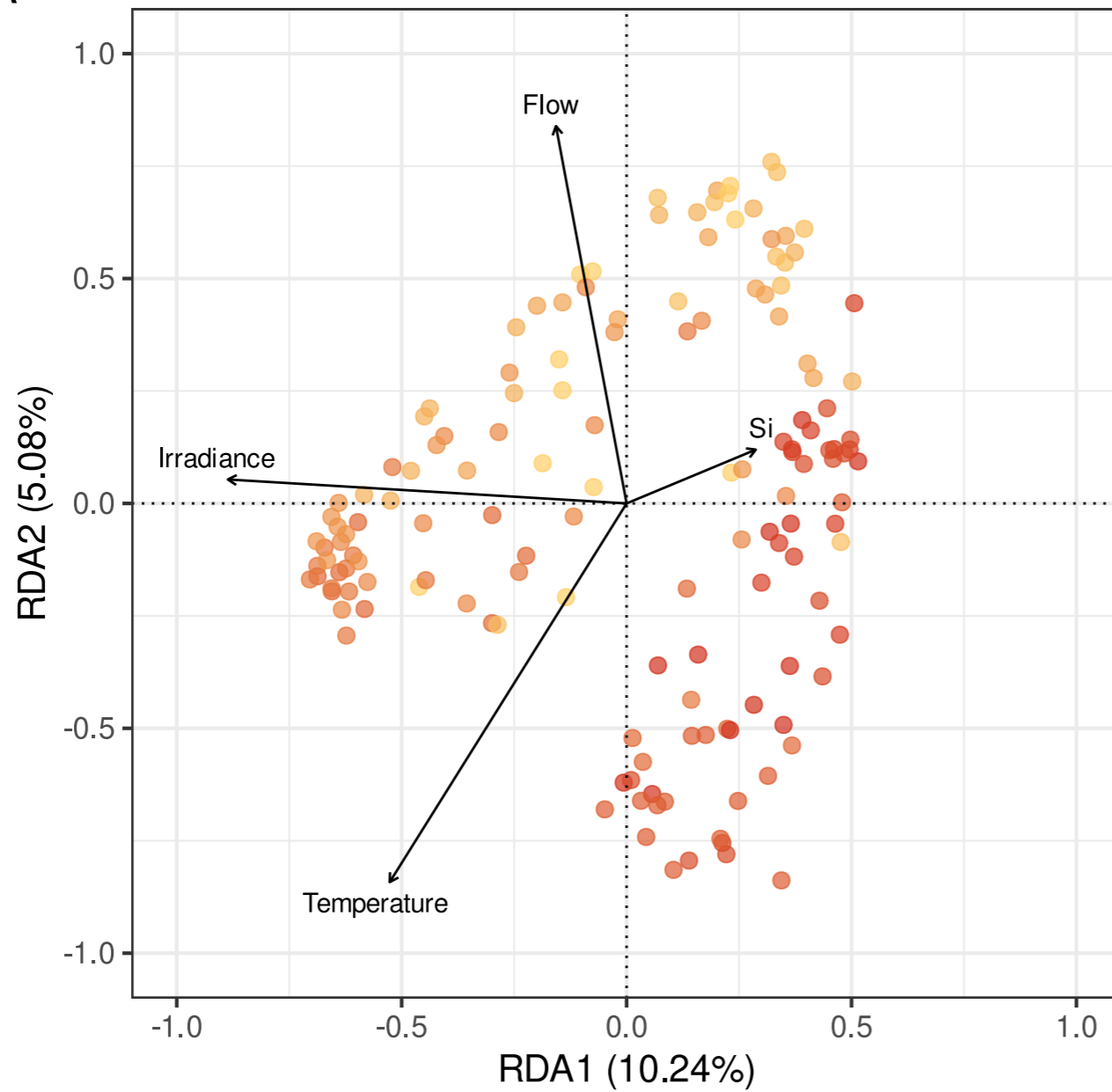

B

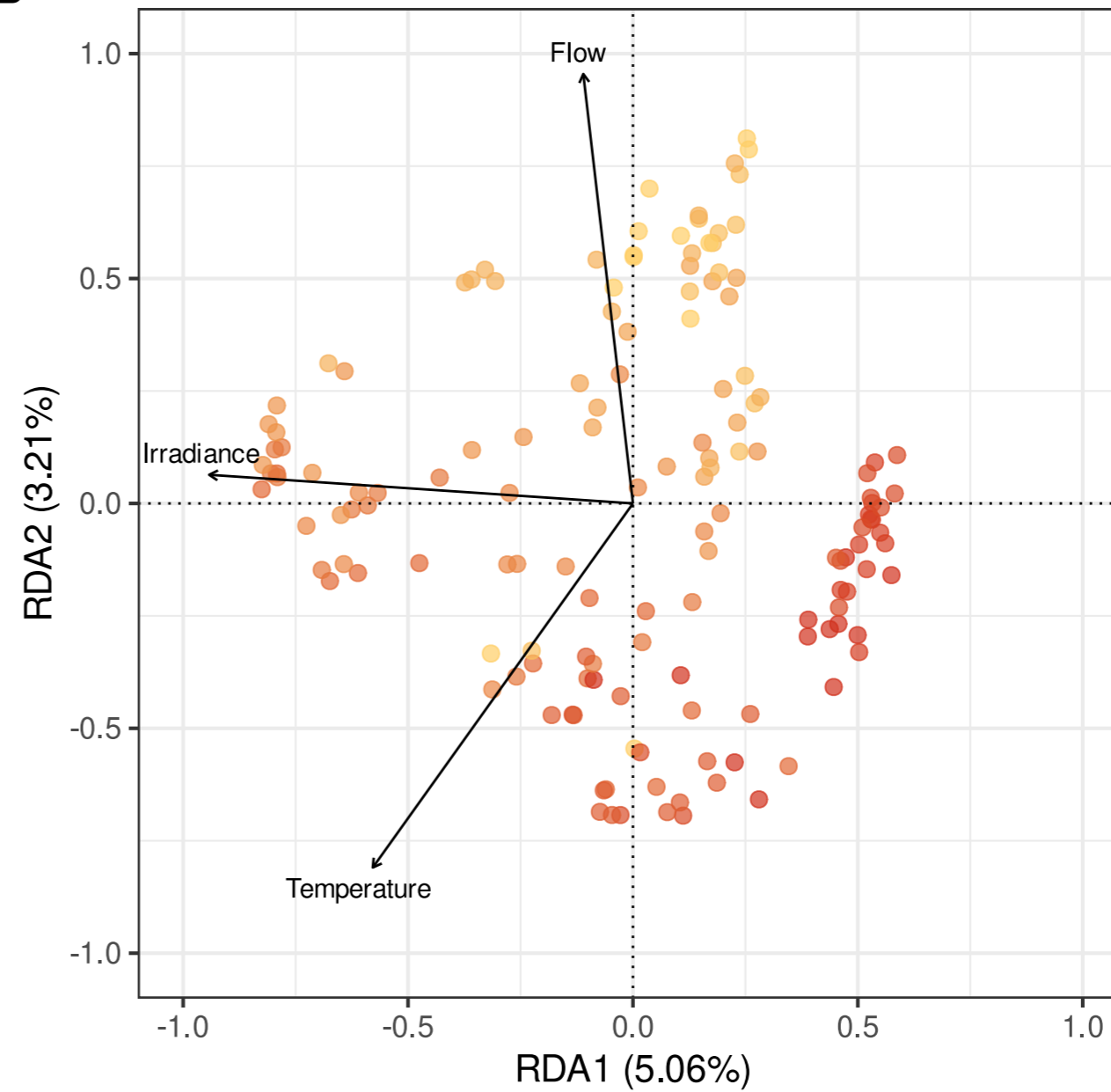

Day

- 19
- 49
- 64
- 107
- 133
- 166
- 196
- 240
- 260
- 285
- 327
- 345

Supplement: Supplementary file 2 — Additional file 2 [file 40793_2025_664_MOESM2_ESM.pdf]

A

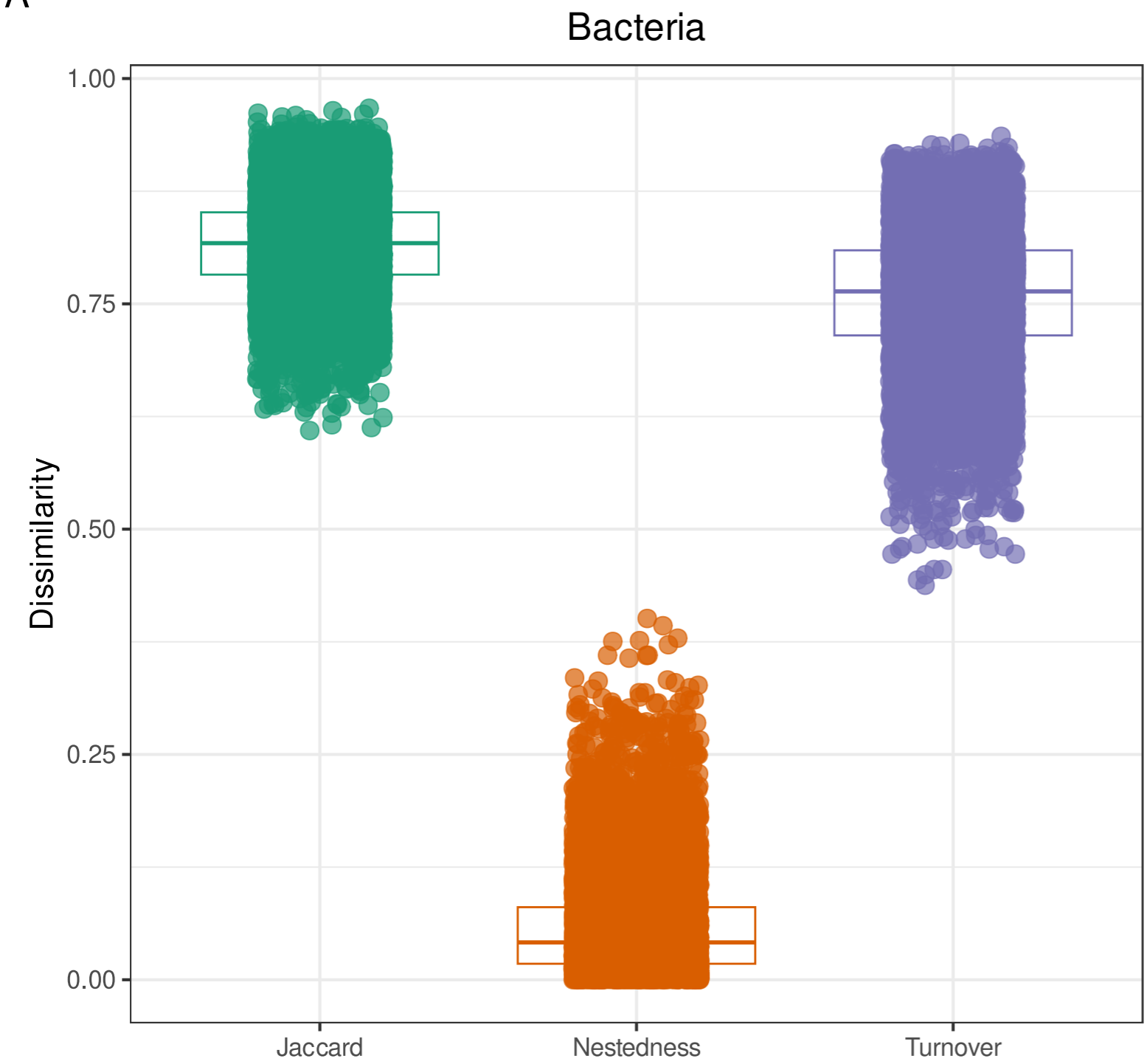

B

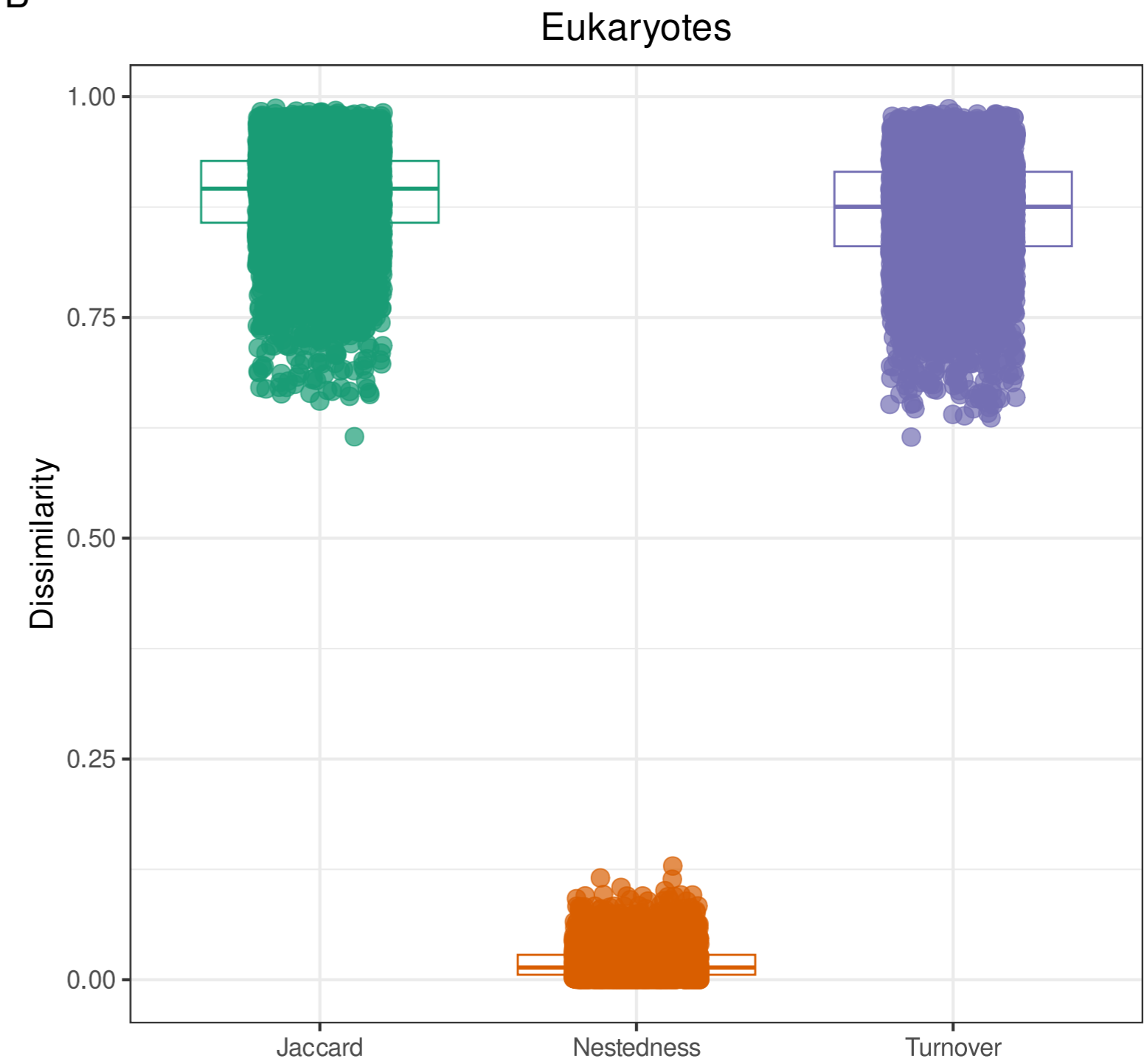

Supplement: Supplementary file 3 — Additional file 3 [file 40793_2025_664_MOESM3_ESM.pdf]
