# Supplementary material for: Together throughout the year: seasonal patterns of bacterial and eukaryotic microbial communities in a macrotidal estuary
Source: Environ Microbiome. 2025 Jan 20;20:8. doi: 10.1186/s40793-025-00664-y (PMC11748528; doi:10.1186/s40793-025-00664-y)
Supplement: Supplementary file 5 — Additional file 5 [file 40793_2025_664_MOESM5_ESM.docx]

**Mantel and partial Mantel tests**

based on Pearson correlations, with 10⁶ permutations, using Bray-Curtis distances for the microbial communities and Euclidean distances for spatial, temporal and physico-chemical matrices. Significant relationships are highlighted in bold.

- All samples (*n* = 147)

|  | r_M_ | *P* value |
| --- | --- | --- |
| Bacteria ~ Eukaryotes | **0.642** | **0.000001** |
| Bacteria ~ Eukaryotes + Chemistry + Space + Time | **0.626** | **0.000001** |
| Bacteria ~ Chemistry | **0.231** | **0.000001** |
| Bacteria ~ Chemistry + Eukaryotes + Space + Time | **0.130** | **0.000001** |
| Bacteria ~ Space | -0.009 | 0.672227 |
| Bacteria ~ Space + Chemistry + Eukaryotes + Time | -0.011 | 0.708002 |
| Bacteria ~ Time | **0.202** | **0.000001** |
| Bacteria ~ Time + Space + Chemistry + Eukaryotes | **0.075** | **0.000259** |
|  |  |  |
| Eukaryotes ~ Bacteria | **0.642** | **0.000001** |
| Eukaryotes ~ Bacteria + Chemistry + Space + Time | **0.623** | **0.000001** |
| Eukaryotes ~ Chemistry | **0.159** | **0.000001** |
| Eukaryotes ~ Chemistry + Bacteria + Space + Time | 0.001 | 0.469289 |
| Eukaryotes ~ Space | -0.003 | 0.558762 |
| Eukaryotes ~ Space + Chemistry + Bacteria + Time | 0.004 | 0.389900 |
| Eukaryotes ~ Time | **0.158** | **0.000001** |
| Eukaryotes ~ Time + Space + Chemistry + Bacteria | **0.034** | **0.026540** |
|  |  |  |
| Chemistry ~ Space | 0.023 | 0.147702 |
| Chemistry ~ Time | **0.399** | **0.000001** |

- Only surface samples (*n* = 100)

|  | r_M_ | *P* value |
| --- | --- | --- |
| Bacteria ~ Eukaryotes | **0.621** | **0.000001** |
| Bacteria ~ Eukaryotes + Chemistry + Space + Time | **0.608** | **0.000001** |
| Bacteria ~ Chemistry | **0.207** | **0.000001** |
| Bacteria ~ Chemistry + Eukaryotes + Space + Time | **0.121** | **0.000063** |
| Bacteria ~ Space | -0.015 | 0.709538 |
| Bacteria ~ Space + Chemistry + Eukaryotes + Time | -0.028 | 0.874836 |
| Bacteria ~ Time | **0.198** | **0.000001** |
| Bacteria ~ Time + Space + Chemistry + Eukaryotes | **0.085** | **0.000950** |
|  |  |  |
| Eukaryotes ~ Bacteria | **0.621** | **0.000001** |
| Eukaryotes ~ Bacteria + Chemistry + Space + Time | **0.608** | **0.000001** |
| Eukaryotes ~ Chemistry | **0.128** | **0.000009** |
| Eukaryotes ~ Chemistry + Bacteria + Space + Time | -0.012 | 0.674250 |
| Eukaryotes ~ Space | 0.014 | 0.255877 |
| Eukaryotes ~ Space + Chemistry + Bacteria + Time | 0.031 | 0.088701 |
| Eukaryotes ~ Time | **0.142** | **0.000002** |
| Eukaryotes ~ Time + Space + Chemistry + Bacteria | 0.028 | 0.099396 |
|  |  |  |
| Chemistry ~ Space | 0.014 | 0.304627 |
| Chemistry ~ Time | **0.395** | **0.000001** |

- Only deeper layer samples (*n* = 47)

|  | r_M_ | *P* value |
| --- | --- | --- |
| Bacteria ~ Eukaryotes | **0.672** | **0.000001** |
| Bacteria ~ Eukaryotes + Chemistry + Space + Time | **0.654** | **0.000001** |
| Bacteria ~ Chemistry | **0.264** | **0.000006** |
| Bacteria ~ Chemistry + Eukaryotes + Space + Time | **0.159** | **0.001614** |
| Bacteria ~ Space | -0.022 | 0.726329 |
| Bacteria ~ Space + Chemistry + Eukaryotes + Time | -0.007 | 0.537782 |
| Bacteria ~ Time | **0.173** | **0.000554** |
| Bacteria ~ Time + Space + Chemistry + Eukaryotes | 0.047 | 0.124013 |
|  |  |  |
| Eukaryotes ~ Bacteria | **0.672** | **0.000001** |
| Eukaryotes ~ Bacteria + Chemistry + Space + Time | **0.654** | **0.000001** |
| Eukaryotes ~ Chemistry | **0.191** | **0.000071** |
| Eukaryotes ~ Chemistry + Bacteria + Space + Time | 0.010 | 0.398940 |
| Eukaryotes ~ Space | -0.026 | 0.760772 |
| Eukaryotes ~ Space + Chemistry + Bacteria + Time | -0.014 | 0.630961 |
| Eukaryotes ~ Time | **0.136** | **0.001263** |
| Eukaryotes ~ Time + Space + Chemistry + Bacteria | 0.022 | 0.287814 |
|  |  |  |
| Chemistry ~ Space | 0.004 | 0.421381 |
| Chemistry ~ Time | **0.378** | **0.000001** |

- Monthly analysis

|  |  | r_M_ | *P* value |
| --- | --- | --- | --- |
| January | Bacteria ~ Space | -0.1995 | 0.9206 |
| February | Bacteria ~ Space | -0.1204 | 0.7623 |
| March | Bacteria ~ Space | -0.1249 | 0.8657 |
| April | Bacteria ~ Space | 0.0822 | 0.2577 |
| May | Bacteria ~ Space | -0.1990 | 0.9476 |
| June | Bacteria ~ Space | -0.0852 | 0.7343 |
| July | Bacteria ~ Space | -0.2220 | 0.9534 |
| August | Bacteria ~ Space | **0.3453** | **0.0107** |
|  | Bacteria ~ Chemistry | **0.3348** | **0.0107** |
|  | Bacteria ~ Space + Chemistry | 0.1259 | 0.1990 |
|  | Space ~ Chemistry | **0.8381** | **0.000002** |
|  | Bacteria ~ Eukaryotes | **0.4650** | **0.0016** |
| September | Bacteria ~ Space | 0.1578 | 0.1335 |
| October | Bacteria ~ Space | **0.3958** | **0.0105** |
|  | Bacteria ~ Chemistry | 0.0900 | 0.1934 |
|  | Bacteria ~ Space + Chemistry | **0.5000** | **0.0036** |
|  | Space ~ Chemistry | **0.7523** | **0.001** |
|  | Bacteria ~ Eukaryotes | **0.7792** | **0.000005** |
| November | Bacteria ~ Space | 0.2331 | 0.0520 |
| December | Bacteria ~ Space | **0.4759** | **0.0008** |
|  | Bacteria ~ Chemistry | **0.4200** | **0.0031** |
|  | Bacteria ~ Space + Chemistry | **0.2480** | **0.038** |
|  | Space ~ Chemistry | **0.8575** | **0.000008** |
|  | Bacteria ~ Eukaryotes | **0.6208** | **0.000007** |
|  |  |  |  |
| January | Eukaryotes ~ Space | -0.2963 | 0.9934 |
| February | Eukaryotes ~ Space | -0.2590 | 0.9754 |
| March | Eukaryotes ~ Space | -0.2449 | 0.9858 |
| April | Eukaryotes ~ Space | -0.0227 | 0.5383 |
| May | Eukaryotes ~ Space | -0.2101 | 0.9574 |
| June | Eukaryotes ~ Space | -0.0045 | 0.4781 |
| July | Eukaryotes ~ Space | -0.1893 | 0.9476 |
| August | Eukaryotes ~ Space | 0.0371 | 0.3167 |
| September | Eukaryotes ~ Space | 0.0754 | 0.2653 |
| October | Eukaryotes ~ Space | **0.4280** | **0.0032** |
|  | Eukaryotes ~ Chemistry | 0.1785 | 0.0853 |
|  | Eukaryotes ~ Space + Chemistry | **0.4531** | **0.0056** |
|  | Space ~ Chemistry | **0.7523** | **0.0001** |
|  | Eukaryotes ~ Bacteria | **0.7792** | **0.000005** |
| November | Eukaryotes ~ Space | -0.0467 | 0.6013 |
| December | Eukaryotes ~ Space | **0.2630** | **0.0308** |
|  | Eukaryotes ~ Chemistry | 0.2140 | 0.0573 |
|  | Eukaryotes ~ Space + Chemistry | 0.1583 | 0.1397 |
|  | Space ~ Chemistry | **0.8575** | **0.000005** |
|  | Eukaryotes ~ Bacteria | **0.6208** | **0.000007** |
|  |  |  |  |
